# Supplementary material for: Molecular Evolution of Far-Red Light-Acclimated Photosystem II
Source: Microorganisms. 2022 Jun 22;10(7):1270. doi: 10.3390/microorganisms10071270 (PMC9325196; doi:10.3390/microorganisms10071270)
Supplement: Supplementary file 1 [file microorganisms-10-01270-s001.zip › microorganisms-1768588-supplementary.pdf]

Supplementary Material for

Molecular evolution of far-red light-acclimated photosystem II

Christopher J. Gisriel[1,†], Tanai Cardona[2,†] Donald A. Bryant[3], and Gary W. Brudvig[1,4,*]

Affiliations:

[1]Department of Chemistry, Yale University, New Haven, CT 06520, USA.

[2]Department of Life Sciences, Imperial College London, London SW7 2AZ, UK

[3]Department of Biochemistry and Molecular Biology, The Pennsylvania State University, University Park, PA 16802, USA.

[4]Department of Molecular Biophysics and Biochemistry, Yale University, New Haven, CT 06520, USA.

[†]These authors contributed equally to this work.

*Correspondence: gary.brudvig@yale.edu

Supplementary Figure S1. Phylogenetic tree of PsbA sequences focused on the FRL sequences and basal clades.

Supplementary Figure S2. Phylogenetic tree of PsbD sequences focused around the FRL sequences and basal clades.

Supplementary Figure S3. Phylogenetic tree of PsbC sequences focused around the FRL sequences and basal clades.

Supplementary Figure S4. Phylogenetic tree of FRL-PsbB and neighboring sequences.

Supplementary Figure S5. Phylogenetic tree of FRL-PsbH and neighboring sequences, and helical prediction.

Supplementary Figure S6. Alignments of ancestral FRL-PSII sequences and selected extant FRL- and VL-specific PSII sequences.

Supplementary Figure S7. Location of the FRL-PsbH2 homology model.

Supplementary Figure S8. Conservation of FRL-PsbD interactions near P_{D2}.

Supplementary Figure S9. Symmetry-related locations of Chl sites 507 and 611.

Supplementary Data S1. Sequence alignments with ancestral sequence reconstructions, and phylogenetic trees used in this study. (external)

Supplementary Figures

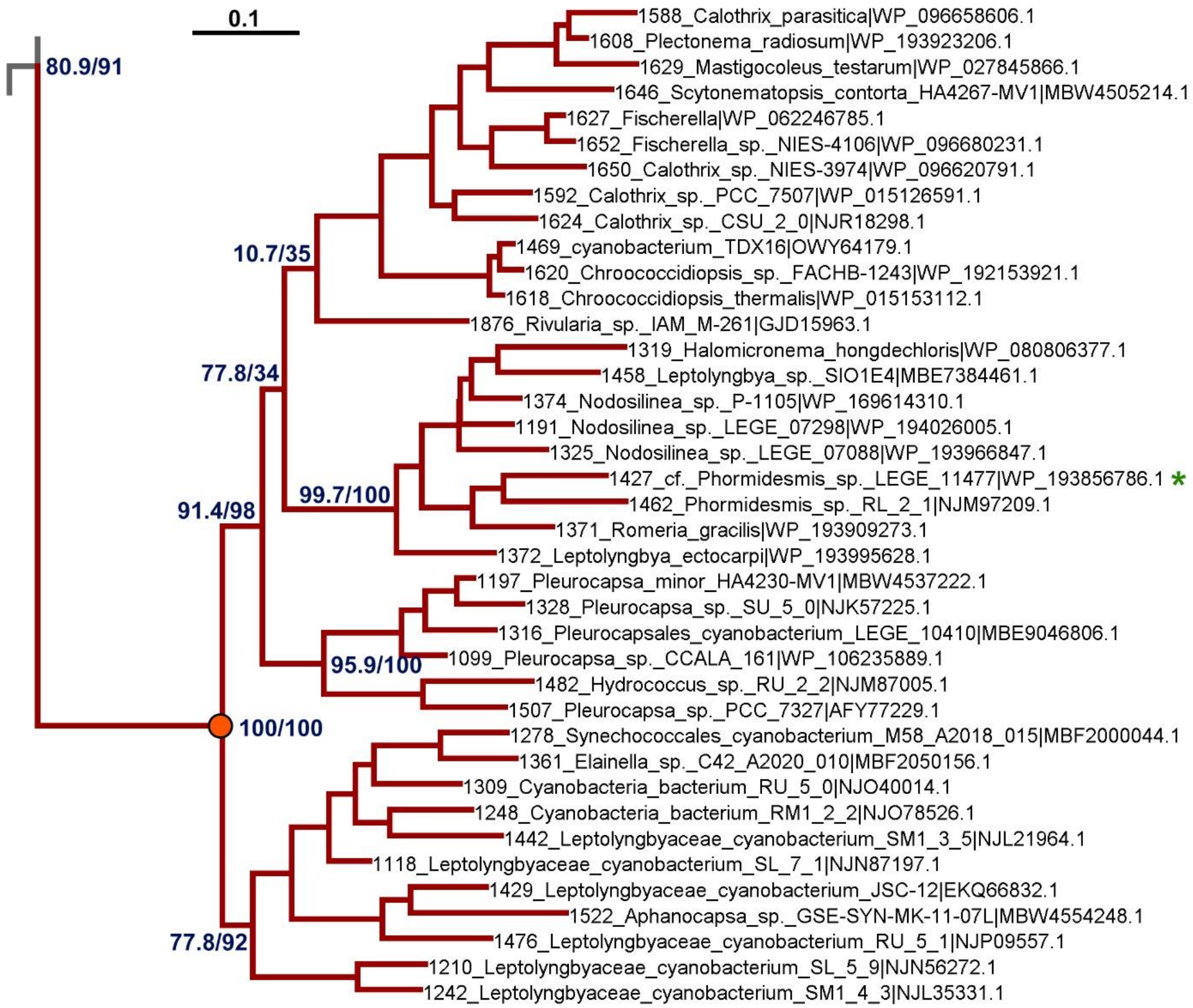

Supplementary Figure S1. Phylogenetic tree of PsbA sequences focused on the FRL sequences and basal clades. The scale bar represents the number of substitutions per site. Blue numbers are branch support values calculated using the average likelihood ratio test and ultrafastbootstrap methods, respectively (aLRT/UFbootstrap). Only those on the major subgroups are shown for clarity. The sequence marked with an asterisk has over 98% sequence identity to that of *Synechococcus* 7335, which is not shown in here due to redundancy curation. The orange circle denotes the most recent common ancestor of FRL sequences used in the FaRLiP response.

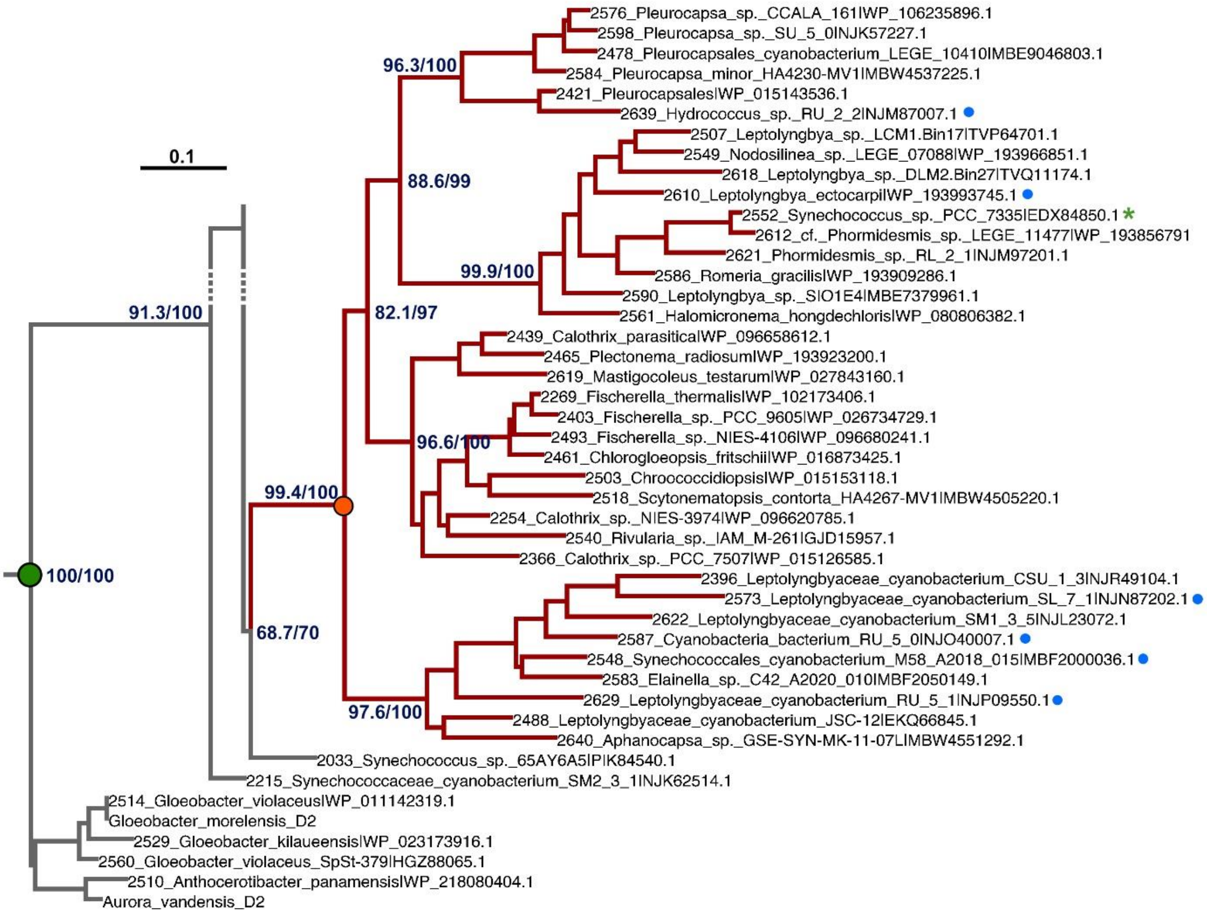

Supplementary Figure S2. Phylogenetic tree of PsbD sequences focused around the FRL sequences and basal clades. The scale bar represents the number of substitutions per site. Blue numbers are support branch support values (aLRT/UFbootstrap). Only those on the major subgroups are shown for clarity. The green circle denotes the most recent common ancestor of cyanobacteria, and the smaller orange circle denotes the most recent common ancestor of FRL sequences used in the FaRLiP response. The sequence with an asterisk is that from *Synechococcus* 7335, for which a structure is available. Sequences marked with blue dots represent independent reversals from PsbD-Tyr161 to Phe161.

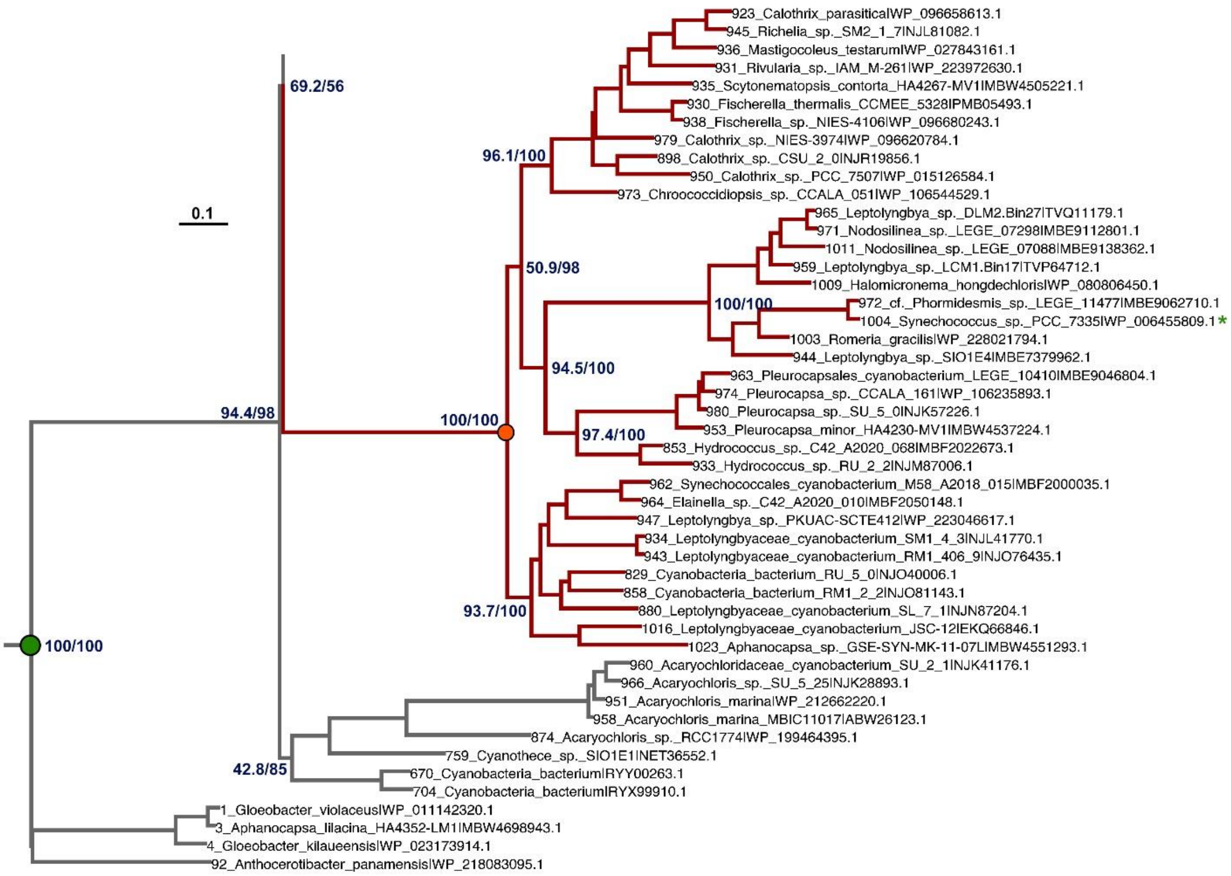

Supplementary Figure S3. Phylogenetic tree of PsbC sequences focused around the FRL sequences and basal clades. The scale bar represents the number of substitutions per site. Blue numbers are branch support values (aLRT/UFbootstrap). Only those on the major subgroups are shown for clarity. The sequence with an asterisk is that from *Synechococcus* 7335, for which a structure is available. The green circle denotes the most recent common ancestor of cyanobacteria, and the smaller orange circle denotes the most recent common ancestor of FRL sequences used in the FaRLiP response.

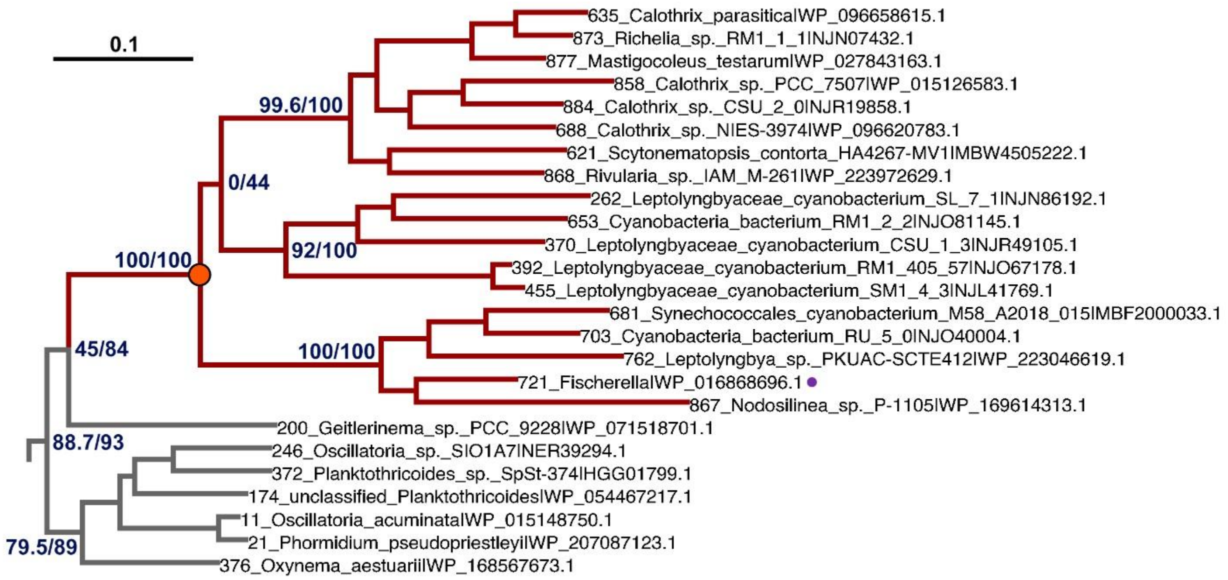

Supplementary Figure S4. Phylogenetic tree of FRL-PsbB (red) and neighboring (gray) sequences. The scale bar represents the number of substitutions per site. Blue numbers are branch support values (aLRT/UFbootstrap). Only those marking the major subgroups are shown for clarity. The orange circle denotes the most recent common ancestor of FRL sequences used in the FaRLiP response. The purple circle highlights the sequence from *Fischerella* (multispecies), which does not group with other heterocystous sequences (e.g., *Calothrix*, *Rivularia*, *Mastigocoleus*).

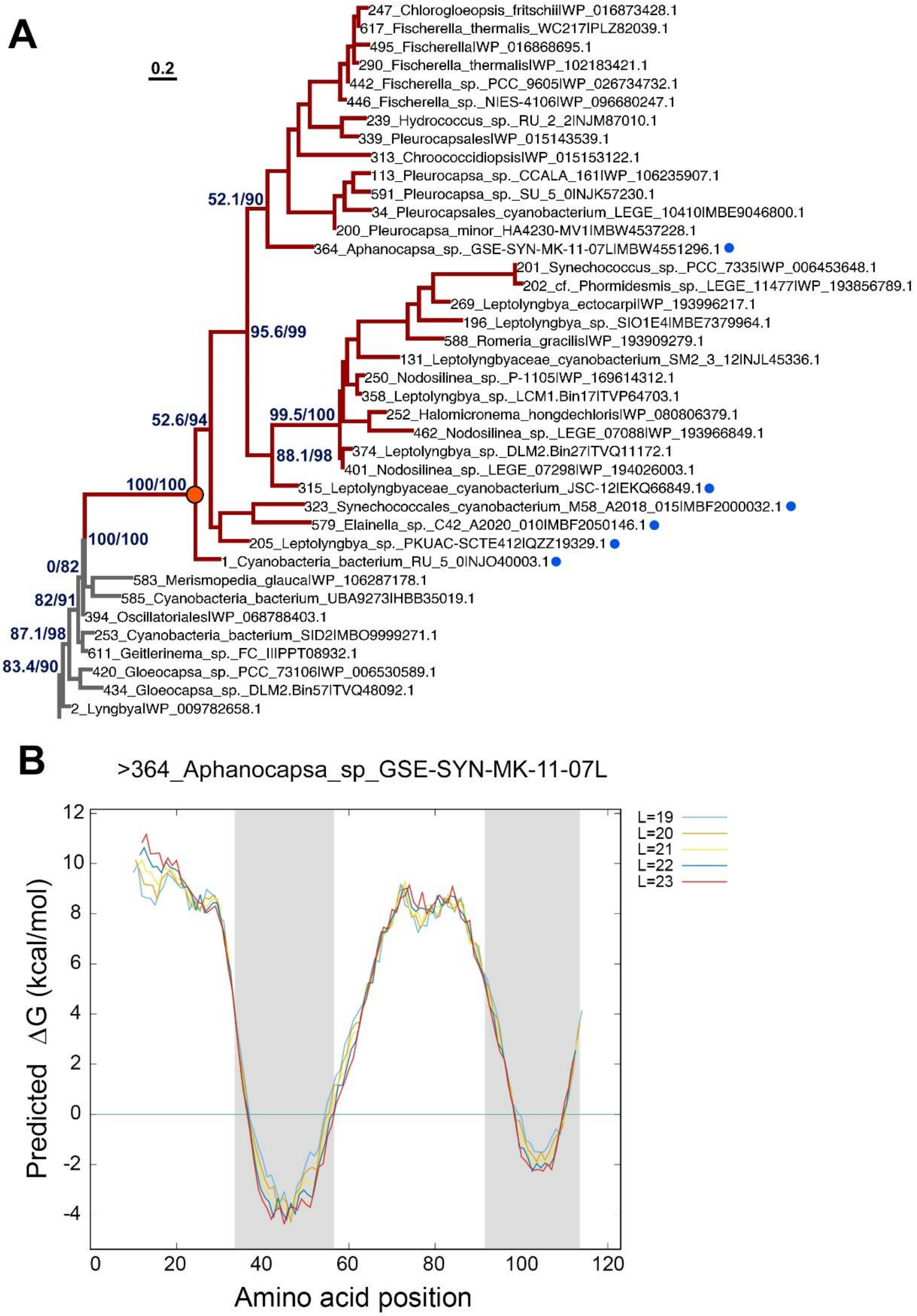

Predicted TM helices

Position		ΔG	Sequence
34-56	(23)	-4.377	TAPLMVVLMLLFLVFLLIIILQIF
92-113	(22)	-2.272	TGTSIFIGLVVFALTCLALIFY

Supplementary Figure S5. Phylogenetic tree of FRL-PsbH and neighboring sequences, and helical prediction. (**A**) shows a phylogenetic tree of FRL-PsbH (red) and neighboring (gray) sequences. The scale bar represents the number of substitutions per site. Blue numbers are branch support values (aLRT/UFbootstrap). Only those on the major subgroups are shown for clarity. The orange circle denotes the most recent common ancestor of FRL sequences used in the FaRLiP response. The blue circle highlights the sequences that contain a second transmembrane helix at the C-terminus. (**B**) shows the prediction of ΔG for transmembrane helix insertion for a representative sequence [1], FRL-PsbH from *Aphanocapsa* GSE. Y axis shows ΔG and X axis the amino acid position in the sequence. L=19 to 23 represent calculations using different transmembrane helix lengths.

PsbA

```
FRL Ancestral          --MTTTLLRKRQSANLWERFCNWVTSTENRLYIGWFGVLMIPLLSVSTCVFVIAFIAAPP 58
FRL Synechococcus 7335 ----MTTLIQRPNISQWERFCQWITSTENRLYIGWFGVLMLPLLGVSITVFVTAFIAAPP 56
FRL Aphanocapsa GSE    ---MNTLIQQGKIVPQWERFCSWVTSTENRLYVGWFGVLMIPLLLVSLSVFAIAFIAAPP 57
FRL Pleurocapsa 7327   ---MTTTLQKREIPNLWEQFCQWVTSTENRLYIGWFGVLMIPLLGVSTTVFVLAIIAAPP 57
FRL Fischerella 7521   --MTTISTRPTSRFPTWDRFCNWVTSTENRLYIGWFGVLMIPLLGVSICVFTIAFIAAPP 58
VL  Synechococcus 7335 ---MTTTLQQRQSASLWEQFCQWVTSTENRLYVGWFGVLMIPTLLAATACFVIAFIAAPP 57
VL  Synechococcus 7335 MVSTTTTLQRSENASLWEQFCQWVTSTENRLYVGWFGVLMIPTLLAATACFVIAFIAAPP 60
VL  Aphanocapsa GSE    ---MTTTLQRRESANLWERFCNWVTSTDNRIYIGWFGVIMVPTLLAATTCFLIAFVAAPP 57
VL  Pleurocapsa 7327   ---MTTTLQTREKASLWEQFCEWITSTNNRIYIGWFGVIMIPTLLTATICYIIAFIAAPP 57
VL  Pleurocapsa 7327   ---MTTTLQQRESASLWEQFCQWVTSTNNRIYIGWFGVIMIPTLLTATACFIIAFIAAPP 57
VL  Pleurocapsa 7327   ---MTTTLQQRESASLWEQFCQWVTSTNNRIYIGWFGVIMIPTLLTATTCFIIAFIAAPP 57
VL  Fischerella 7521   ---MTTTLQRRASGNVWERFCDWVTSTENRLYIGWFGVLMIPTLLAATTCFIIAFIAAPP 57
VL  Fischerella 7521   ---MTATLQRAQSANVWERFCNWITSTENRLYIGWFGVLMIPTLLAATTCFIIAFIAAPP 57
VL  Fischerella 7521   ---MTTTLTRGESGSLWDRFCEWITSTNNRLYIGWFGVLMIPTLLTATICFIIAFIAAPP 57
                                       *::**.*:***:**:*:*****:*:* *  .:   :  *::****

FRL Ancestral          VDIDGIREPVSGSLLYGNNIITAAVVPSSNAIGLHFYPIWEAASMDEWLYNGGPYQMIAF 118
FRL Synechococcus 7335 VDIDGIREPLSGSLLYGNNIITAAVVPTSNAIGLHFYPIWEAATLDEWLYNGGPYQMIAF 116
FRL Aphanocapsa GSE    VDIDGIREPVCGSLLCGNNIITGAVVPSSNAIGLHFYPIWDAASIDEWLYNGGPYQMIGA 117
FRL Pleurocapsa 7327   VDMDGLREPITGSLLYGNNIITAAVVPTSNAIGLHFYPIWEAASMDEWLYNGGPYQMIAL 117
FRL Fischerella 7521   VDIDGIREPVSGSLLYGNNIITAAVVPMSNAIGLHFYPIWEAASMDEWLYNGGPYQMIGF 118
VL  Synechococcus 7335 VDIDGIREPVAGSLMYGNNIISGAVVPSSNAIGLHFYPIWEAASLDEWLYNGGPYQLVIF 117
VL  Synechococcus 7335 VDIDGIREPVAGSLMYGNNIISGAVVPSSNAIGLHFYPIWEAASLDEWLYNGGPYQLVIF 120
VL  Aphanocapsa GSE    VDIDGIREPVAGSLIFGNNIITGAVIPSSNAIGLHFYPIWEAASLDEWLYNGGPYQLIIF 117
VL  Pleurocapsa 7327   VDIDGIREPVSGSLLYGNNIISGAVVPSSNAIGLHFYPIWEAASLDEWLYNGGPYQLILF 117
VL  Pleurocapsa 7327   VDIDGIREPVAGSLLYGNNIISGAVVPSSNAIGLHFYPIWEAASLDEWLYNGGPYQLTIF 117
VL  Pleurocapsa 7327   VDIDGIREPVAGSLLYGNNIISGAVVPSSNAIGLHFYPIWEAASLDEWLYNGGPYQLTIF 117
VL  Fischerella 7521   VDIDGIREPVAGSLLYGNNIISGAVVPSSNAIGLHFYPIWEAASLDEWLYNGGPYQLVIF 117
VL  Fischerella 7521   VDIDGIREPVAGSLLYGNNIISGAVVPSSNAIGLHFYPVWEAASLDEWLYNGGPYQLVIF 117
VL  Fischerella 7521   VDIDGIREPVSGSLLYGNNIISGAIVPTSNAIGLHFYPIWEASSLDEWLYNGGPYELIVF 117
                       **:**:***: ***: *****:.*::* **********:*:*:::**********::

FRL Ancestral          HYIPALCCYMGREWELSYRLGMRPWICVAYSAPLAATTSVFLIYPIGQGSFSDGLPMGIS 178
FRL Synechococcus 7335 HYIPALLCYLGREWELSYRLGMRPWICIAYSAPVAATISVFLIYPIGQGSFSDGLPMGIS 176
FRL Aphanocapsa GSE    HYIPALACYMGRQWELSYRLGMRPWICVAYSAPLISTTSVFWIYPIGQGSFSDGLPMGIS 177
FRL Pleurocapsa 7327   HYVPALCCYLGREWELSYRLGMRPWICVAFSAPLAATTSVFLIYPLGQGSFADGLPMSIS 177
FRL Fischerella 7521   HYIPALACYMGREWELSYRLGMRPWIAVAYSAPLAATTSVFLIYPIGQGSFSDGLPMGIS 178
VL  Synechococcus 7335 HFLIGVFCYMGREWELSYRLGMRPWICVAYSAPVAAATAVFLIYPLGQGSFSDGMPLGIS 177
VL  Synechococcus 7335 HFLIGVFCYMGREWELSYRLGMRPWICVAYSAPVAAATAVFLIYPLGQGSFSDGMPLGIS 180
VL  Aphanocapsa GSE    HFLLGCACYMGRQWELSYRLGMRPWICVAYSAPLASATAVFLIYPIGQGSFSDGMPLGIS 177
VL  Pleurocapsa 7327   HFLLGICCWMGRQWELSYRLGMRPWICVAYSAPLASAFAVFLIYPIGQGSFSDGMPLGIS 177
VL  Pleurocapsa 7327   HFLLGVFCYMGRQWELSYRLGMRPWICVAYSAPVSAATAVFLIYPIGQGSFSDGMPLGIS 177
VL  Pleurocapsa 7327   HFLLGVFCYMGRQWELSYRLGMRPWICVAYSAPVSAATAVFLIYPIGQGSFSDGMPLGIS 177
VL  Fischerella 7521   HFLIGCFCYMGRQWELSYRLGMRPWICVAYSAPLASATAVFLIYPLGQGSFSDGMPLGIS 177
VL  Fischerella 7521   HFLIGVFCYMGREWELSYRLGMRPWIAVAYSAPVAAATAVFLIYPLGQGSFSDGMPLGIS 177
VL  Fischerella 7521   HFLIGIFCWMGRQWELSYRLGMRPWICVAYSAPVAAATSVFLIYPIGQGSFSDGMPLGIS 177
                       *::  .   *::**:*************.:*:***: :: :** ***:*****:**:*:.**

FRL Ancestral          GTFNFMFVFQAEHNILMHPFHMLGVAGVFGGSLFCAMHGSLVTSSLVRETTEQESQNYGY 238
FRL Synechococcus 7335 GTFNFMFVFQAEHNILMHPFHMLGVAGVLGGSLFCAMHGSLVTSSLVRETSDSQSQNEGY 236
FRL Aphanocapsa GSE    GTFNFMFVFQAEHNILMHPFHMLGVIGVFGGSLFCAMHGSLVTSSLVRETTENESANLGY 237
FRL Pleurocapsa 7327   GTFNFMFVFQAEHNILMHPFHMLGVAGVFGGAFFCAMHGSLVTSSLIRETSENESQNYGY 237
FRL Fischerella 7521   GTFNFMFVFQAEHNILMHPLHMIGVAGVLGGSLFCAMHGSLVTSSLIRETTELESQNYGY 238
VL  Synechococcus 7335 GTFNFMLVFQAEHNILMHPFHMLGVAGVFGGSLFSAMHGSLVTSSLVRETTETESQNYGY 237
VL  Synechococcus 7335 GTFNFMLVFQAEHNILMHPFHMLGVAGVFGGSLFSAMHGSLVTSSLVRETTETESQNYGY 240
VL  Aphanocapsa GSE    GTFNFMIVFQAEHNILMHPFHMLGVAGVFGGSLFSAMHGSLVTSSLIRETTEVESANYGY 237
VL  Pleurocapsa 7327   GTFNFMFVFQAEHNILMHPFHMLGVAGVFGGSLFCAMHGSLVTSSLVRETSENESQNYGY 237
VL  Pleurocapsa 7327   GTFNFMFVFQAEHNILMHPFHMLGVAGVFGGSLFSAMHGSLVTSSLVRETTETESQNYGY 237
VL  Pleurocapsa 7327   GTFNFMFVFQAEHNILMHPFHMLGVAGVFGGSLFSAMHGSLVTSSLVRETTETESQNYGY 237
VL  Fischerella 7521   GTFNFMIVFQAEHNILMHPFHQLGVAGVFGGSLFCAMHGSLVTSSLVRETTETESQNYGY 237
VL  Fischerella 7521   GTFNFMLVFQAEHNILMHPFHQLGVAGVFGGSLFSAMHGSLVTSSLVRETTETESQNYGY 237
VL  Fischerella 7521   GTFNFMLVFQAEHNILMHPFHQLGVAGVFGGSLFCAMHGSLVTSSLVRETTENESVNYGY 237
                       ******:************:* :** **:**::*.***********:***:: :* * **

FRL Ancestral          KFGQEEETYNIVAAHGYFGRLIFQYNEKNNSRSLHFFLAAWPVVCIWFTALGISTMAFNL 298
FRL Synechococcus 7335 KFGQEEETYNILAAHGYFGRLIFQYASFNNSRQLHFFLAAWPVVCIWFVALGISTMAFNL 296
FRL Aphanocapsa GSE    KFGQQNETYNIVAAHGYFGRLIFQYASFNNSRSLHFFLGAFPVVCIWCTALGICTMGFNL 297
FRL Pleurocapsa 7327   KFGQEEETYNIVAAHGYFGRLIFQYASFNNSRNLHFFLAAWPVICIWFTALGICVMAFNL 297
FRL Fischerella 7521   KFGQEQETYNIVAAHGYFGRLIFQYASFNNSRSLHFFLAAWPVICIWGTAIGISTMAFNL 298
```

```
VL  Synechococcus 7335 KFGQEEETYNIVAAHGYFGRLIFQYASFNNSRSLHFLLGAWPVVGIWFTALGISTMAFNL 297
VL  Synechococcus 7335 KFGQEEETYNIVAAHGYFGRLIFQYASFNNSRSLHFLLGAWPVVGIWFTALGISTMAFNL 300
VL  Aphanocapsa GSE    KFGQEQETYNIVAAHGYFGRLIFQYASFNNSRSLHFLLGAWPVVGIWFTALGISTMAFNL 297
VL  Pleurocapsa 7327   KFGQEEETYNIVAAHGYFGRLIFQYASFNNSRSLHFFLGAWPVVCIWFTAMGVSTMAFNL 297
VL  Pleurocapsa 7327   KFGQEEETYNIVAAHGYFGRLIFQYASFNNSRALHFFLGAWPVIGIWFTAMGISTMAFNL 297
VL  Pleurocapsa 7327   KFGQEEETYNIVAAHGYFGRLIFQYASFNNSRALHFFLGAWPVIGIWFTAMGISTMAFNL 297
VL  Fischerella 7521   KFGQEEETYNIVAAHGYFGRLIFQYASFNNSRSLHFFLAAWPVVGIWFTALGISTMAFNL 297
VL  Fischerella 7521   KFGQEEETYNIVAAHGYFGRLIFQYASFNNSRSLHFFLAAWPVIGIWFTALGISTMAFNL 297
VL  Fischerella 7521   KFGQEQETYNIVAAHGYFGRLIWQYASFNNSRSLHFFLAAWPVVCIWFTALGISTMAFNL 297
                       ****::*****:**********:** . **** ***:*.*:**: ** .*:*:..*.***

FRL Ancestral          NGFNFNQSVLDSQGRVINTWADVLNRANLGFEVMHERNAHNFPLDLAGGEAVPVALRAPA 358
FRL Synechococcus 7335 NGFNFNHSVLDSQGRVLPSWADVVNRASLGFEVMHERNAHNFPLDLASGESVQVAMRAPH 356
FRL Aphanocapsa GSE    NGFNFNHSVLDSQGTVINTWADVLNRANLGFEVMHERNAHNFPLDLAGAEAVPVALSAPA 357
FRL Pleurocapsa 7327   NGFNFNNSIHDAQGRVLPSWADVINQANLGFEVMHERNAHNFPLDLASGEAIPVALRAPA 357
FRL Fischerella 7521   NGFNFNNSILDSQGRVLPSWADVLNRANLGFEVMHERNAHNFPLDLAGGEAVPVAITAPS 358
VL  Synechococcus 7335 NGFNFNQSIIDSQGRVIGSWADVLNRANLGMEVMHERNAHNFPLDLAAGEAAPVALTAPS 357
VL  Synechococcus 7335 NGFNFNQSIIDSQGRVIGSWADVLNRANLGMEVMHERNAHNFPLDLAAGEAAPVALTAPS 360
VL  Aphanocapsa GSE    NGFNFNHSVLDAQGNVINTWADLINRANLGMEVMHERNAHNFPLDLAAGEVAPVALTAPA 357
VL  Pleurocapsa 7327   NGFNFNQSVLDSQGRVISTWADVLNRANLGFEVMHERNAHNFPLDLASGEATPIALTAPS 357
VL  Pleurocapsa 7327   NGFNFNQSILDSQGRVINTWADVLNRANLGFEVMHERNAHNFPLDLASGEMAPVALSAPA 357
VL  Pleurocapsa 7327   NGFNFNQSILDSQGRVINTWADVLNRANLGFEVMHERNAHNFPLDLASGEMAPVALSAPA 357
VL  Fischerella 7521   NGFNFNQSVIDSQGRVINTWADIINRANLGMEVMHERNAHNFPLDLASGDVAPVALTAPA 357
VL  Fischerella 7521   NGFNFNQSVIDSQGRVINTWADIINRANLGMEVMHERNAHNFPLDLASGDVAPVALTAPA 357
VL  Fischerella 7521   NGFNFNQSILDSHGRIVNTWADILNRANLGMEVMHERNAHNFPLDLASGEAVPVAMNAPA 357
                       ******:*: *::* :: :***::*:*.**:****************..:   :*: **

FRL Ancestral          IAS------ 361
FRL Synechococcus 7335 IGA------ 359
FRL Aphanocapsa GSE    IPSQSLASL 366
FRL Pleurocapsa 7327   IAS------ 360
FRL Fischerella 7521   ITA------ 361
VL  Synechococcus 7335 INA------ 360
VL  Synechococcus 7335 INA------ 363
VL  Aphanocapsa GSE    IHG------ 360
VL  Pleurocapsa 7327   IEG------ 360
VL  Pleurocapsa 7327   ING------ 360
VL  Pleurocapsa 7327   ING------ 360
VL  Fischerella 7521   ING------ 360
VL  Fischerella 7521   ING------ 360
VL  Fischerella 7521   IHG------ 360
                       * .
```

PsbB

```
FRL Ancestral          MGLPWYRVHTVVLNDPGRLIAVHLMHTALTAGWAGSMALYELALFDPSDPVLNPMWRQGM 60
FRL Synechococcus 7335 MGLPWYRVHTSVLNDPGRLIAVHIMHNALCAGFAGSMLLFELALFDPSDPVLNPMWRQGC 60
FRL Aphanocapsa GSE    MGLPWYRVHTVVLNDPGRLLAVHLMHNALCAGFAGSMLLYELALFDPTDPVLNPMWRQGC 60
FRL Pleurocapsa 7327   MGLPWYRVHTVVLNDPGRLIAVHLMHNALCAGFAGSMLLFELALYDPSDPVLNPMWRQGC 60
FRL Fischerella 7521   MGLPWYRVHTVVLNDPGRLIAVHLMHNALCAGFAGSMLLFELALYDPSDPVLNPMWRQGC 60
VL  Synechococcus 7335 MGLPWYRVHTVVLNDPGRLISVHLMHTALVAGWAGSMALFELATFDPSDPVLNPMWRQGM 60
VL  Aphanocapsa GSE    MGLPWYRVHTVLINDPGRLIATHLMHTALVAGWAGSMALYELAVFDPSDPVLNPMWRQGM 60
VL  Pleurocapsa 7327   MGLPWYRVHTVVLNDPGRLISVHLMHTALVAGWAGSMALYELAIFDPSDPVLNPMWRQGM 60
VL  Fischerella 7521   MGLPWYRVHTVVLNDPGRLISVHLMHTALVAGWAGSMALYELAIYDPSDPVLNPMWRQGM 60
                       ********** ::******::.*:**.** **:**** *:*** :**:***********

FRL Ancestral          FVMPFMARLGVTNSWGGWSITGEEATDPGFWTFETVAVAHIILSGLLFLAACWHWVYWDL 120
FRL Synechococcus 7335 FLMPFVSRLGVVNSWQGWSVTGETFTNPGFWTFETVAIAHIIFSGLSFLAACWHWVYWDV 120
FRL Aphanocapsa GSE    FLMPFVARLGVVNSWQGWSITGGTVNDPGFWTLESVAIAHIIFSGLEFLAAVWHWNYWNI 120
FRL Pleurocapsa 7327   FLMPFVARLGVTNSWQGWSITGETFDDPGFWTFETVAIAHIVFSGLEFLAAIWHWVNWDL 120
FRL Fischerella 7521   FLMPFVARLGVTNSWQGWSVTGETFADPGFWTFETVAIAHIVFSGLEFLAACWHWFYWDL 120
VL  Synechococcus 7335 FVLPFMTRLGVTGSWGGWDVTGATGISPGFWSFEGVALAHIVLSGLLFLAACWHWVFWDL 120
VL  Aphanocapsa GSE    FVLPFMARLGITGSWGSWSITGETAVNPGFWSFEGVALAHIVLSGLLFLAAIWHWVNWDL 120
VL  Pleurocapsa 7327   FVLPFMARLGVTGSWGGWSVTGETGVNPGFWSFEGVAAAHIVLSGLLFLAAVWHWVYWDL 120
VL  Fischerella 7521   FVLPFMARLGVVGSWGGWNVTGAANYDPGFWSFEGVAAAHIVLSGLLFLAAVWHWVYWDL 120
                       *::**::***:..** .*.:**    .****::* ** ***::*** **** ***  *::

FRL Ancestral          ELFTDSRTGEPALDLPKIFGIHLFLAGLLCFGFGAFHLTGIFGPGMWVSDPYGLTGHVQP 180
FRL Synechococcus 7335 ATFFDPKTDEPVIDLPKVFGIHLTLAGILCFGFGAFHLTGLFGPGMWVSDPLGLTGHIQG 180
FRL Aphanocapsa GSE    STFFDPKTNKPILDLPRIFGIHLFLAGLACFGFGAFHLTGIFGPGMWVSDPFGLTGHVQG 180
FRL Pleurocapsa 7327   VTFFDEKTGEPTLDLPRIFGIHLFLAGLLCFGFGAFHLTGIFGPGMWVSDPYGLTGHVQG 180
FRL Fischerella 7521   ATFFDSKTGEPTLDLPKIFGIHLFLAGLLCFGFGAFHLTGIFGPGMWVSDPYGLTGHVQG 180
```

```
VL  Synechococcus 7335 ELFRDPRTGEPALDLPKMFGIHLFLSGLLCFGFGAFHLTGLWGPGMWVSDPYGLTGHVQG 180
VL  Aphanocapsa GSE    ELFRDPRTGEPALDLPKMFGIHLFLSGLLCFSFGAFHLTGLFGPGMWVSDPYGLTGSIQP 180
VL  Pleurocapsa 7327   ELFTDPRTGEPALDLPKMFGIHLFLSGLLCFGFGAFHLTGLWGPGMWVSDPYGLTGHVQP 180
VL  Fischerella 7521   ELFQDPRTGEPALDLPKMFGIHLFLSGLLCFGFGAFHLTGLWGPGMWVSDAYGLTGHIAP 180
                       * * :*.:* :***::***** *:*: **.********::********  **** :

FRL Ancestral          VAPEWGPDGFNPFNPGGVVAHHIAAGIVGIIGGLFHITVRPPENLYRALRMGNIETVLAS 240
FRL Synechococcus 7335 VAPEWGAAGFDPHNPGGVVAHHIALGIVAIIGGLFHIFVRPPEYLYKGLRMGNIEGTLAS 240
FRL Aphanocapsa GSE    VAPDWGASGFNPFNPGGVVAHHIAAGIVGIVGGLFHMNVRPSENLYKGLRMGNLETVLAS 240
FRL Pleurocapsa 7327   VAPVWGPEGFNPYNPGGVVAHHIAAGIVGIIGGLFHIVVRPSEGLYRLLRMGNIEGVLAS 240
FRL Fischerella 7521   VAPVWGPEGFNPQNPGGVVAHHIAAGIVGIIGGLFHIVVRPPEVLYRGLRMGNIETVLAS 240
VL  Synechococcus 7335 VAPEWGPAGFNPFNPGGVVAHHIAAGIVGIVAGLFHLTVRPPQRLYKALRMGNIETVLSS 240
VL  Aphanocapsa GSE    VAPAWGPDGFNPFNPGGIVAHHIAAGVVGIIAGLFHLIVRPPQRLYKALRMGNIETVLSS 240
VL  Pleurocapsa 7327   VAPEWGPAGFNPFNPGGVVAHHIAAGIVGIIAGLFHLTVRPPERLYRALRMGNIETVLSS 240
VL  Fischerella 7521   VAPEWGPDGFNPFNPGGVVAHHIAAGIVGIIAGLFHLSVRPPERLYKALRMGNIETVLSS 240
                       *** **  **:* ****:****** *:*.*:.****: ***  : **: *****:* .*:*

FRL Ancestral          ALATFFFAGFVVAGTMWYGSATTPIELFGPTRYQWDSGYFQQEIDRRVQANLAEGKSLSE 300
FRL Synechococcus 7335 GLAVFFSGAFIAAGTMWYGTATTPIELWGPTRYQWDQGFFQQAISRQVKASISDGKSPSE 300
FRL Aphanocapsa GSE    GLATFFFAAFVASGSMWYGTATTPVELWGPTRFQWDKGYFKHEIDRRVQASLAEGKSLSQ 300
FRL Pleurocapsa 7327   SLAVFFFAGFIASASMWYGTATTPIELWGPTRYQWDRGYFQQEIDRRVQAGLAEGKSLNQ 300
FRL Fischerella 7521   ALATFFFAGFVAAGSMWYGTATTPIELWGPTRYQWDQNYFKQEIDRRVQAGLDEGKTLSQ 300
VL  Synechococcus 7335 SIAAVFFAAFIVAGTMWYGSATTPIELFGPTRYQWDGSYFAEEIDRRVQRDIANGASEED 300
VL  Aphanocapsa GSE    SIAAVFFAAFVVAGTMWYGSAATPIELFGPTRYQWDGSYFNQEINRRVQTSLAQGASLSE 300
VL  Pleurocapsa 7327   SIAAVFFAAFVVAGTMWYGSATTPIELFGPTRYQWDKGYFQQEIQRRVEANLAAGDTLSE 300
VL  Fischerella 7521   SIAAVFFAAFVVAGTMWYGSATTPIELFGPTRYQWDQGYFKQEIQRRVQASLASGANLSE 300
                       .:*..*  ..*:.:.:****:*:**:**:****:***  .:* . *.*:*: .:   *  . .:

FRL Ancestral          AWSSIPEKLAFYDYVGNSPAKGGLFRVGPMTKGDGIAQSWLGHPVFQDAEGRELTVRRMP 360
FRL Synechococcus 7335 AWSEIPTKLAFYDYIGNSPAKGGLFRVGRMVDGDGLPTGWLGHPVFKDGEGRELTVRRMP 360
FRL Aphanocapsa GSE    AWSAIPEKLAFYDYTGNNPAKGGLFRVGRMVDGDGVAQDWLGHPVFTDQEGRELTVRRMP 360
FRL Pleurocapsa 7327   AWSAIPPKLAFYDYIGNSPAKGGLFRVGRMVDGDGLATGWLGHPVFKDGEGRELTVRRMP 360
FRL Fischerella 7521   SWSAIPEKLAFYDYIGNNPAKGGLFRVGRMVDGDGVAQSWLGHPVFKDREGRELTVRRMP 360
VL  Synechococcus 7335 AYAAIPEKLAFYDYVGNSPAKGGLFRVGPMNTGDGIATAWLGHPVFHDGDGRELTVRRLP 360
VL  Aphanocapsa GSE    AWSAIPEKLAFYDYIGNNPSKGGLFRTGAMDNGDGIAKGWIGHATFRDAEGTELFVRRMP 360
VL  Pleurocapsa 7327   AWSKIPEKLAFYDYVGNSPAKGGLFRTGAMNSGDGIARAWLGHAVFRDGEGRELSVRRMP 360
VL  Fischerella 7521   AWSQIPEKLAFYDYVGNSPAKGGLFRTGPMVKGDGIAQSWDGHPVFKDAEGRELEVRRLP 360
                       ::: ** ******* **.*:******.* *  ***:    * ** .* * :* ** ***:*

FRL Ancestral          NFFETFPVVLTDKDGIVRADIPFRRAESKYSFEQTGVTVSFYGGELDGQTFTDPATVKKY 420
FRL Synechococcus 7335 NFFENFPVVLFDQDGIVRADIPFRQAESKYGIEQTGVTVSFYGGELDGQTFSDPKDVKKY 420
FRL Aphanocapsa GSE    SFFENFPVIFVDNDGVVRADIPFRRAAAKYSVEQTGVNVTFYGGELDGQTFTDPGMVKRY 420
FRL Pleurocapsa 7327   NFFETFPVVMTDKDGVVRADIPFRRTDAKYSIDQTGVTVSFYGGLLDGQTISDPALVKQY 420
FRL Fischerella 7521   TFFETFPVVLTDKDGVVRADIPFQRAEAKYSFEQTGVNVSFFGGILDGQTFTDPMTVKKY 420
VL  Synechococcus 7335 NFFETFPVVLVDKDDNLRADIPFRRAESKYSFEQTGVVVDFYGGQLDGQHITDPAAVKRY 420
VL  Aphanocapsa GSE    NFFETFPVILTDKDGDVRADIPFRRAESKYSFEQQGVNVSFYGGELDGQTFKDAPTVKKY 420
VL  Pleurocapsa 7327   NFFETFPVVLTDSDGIVRADIPFRRAESKTSIEQTGTKVSFYGGILDGQTFSDPATVKQF 420
VL  Fischerella 7521   NFFETFPVILTDKDGIVRADIPFRRAESQNSFEQTGVTVSFYGGNLDGQTFTDPADVKKW 420
                       .***.***:: *.*. :******::: :: ..:* *. * *:** **** :.*   **::

FRL Ancestral          ARKAQLGEPFEFDRQILNSDGVFRTSTRGWFAFAHACFALLWFFGHIWHGSRTLFRDVFA 480
FRL Synechococcus 7335 ARRAQLGEPFEFDRSVYDSDGLFRTSNRGFFAFFHVIFGLLWFFGHIWHGLRALFQDVFS 480
FRL Aphanocapsa GSE    ARQAQLGEVFNFNRETYNSDGVFRTSNRGFFAFFHACFALVWFFGHLWHGSRTLFRDVFA 480
FRL Pleurocapsa 7327   ARKAQLGEPFDFDRQAYNSDGVFRTSNRGWFAFFHACFALVWFFGHVWHGARTLFRDVFA 480
FRL Fischerella 7521   ARQAQLGEPFKFDRTIHNSDGVFRTSNRGFFAFFHTCFALVWFFGHIWHGSRTIYRDVFA 480
VL  Synechococcus 7335 ARKAQLGEPFSFDRETLDSDGVFRSSPRGWFTYGHAVFALLFFFGHIWHGARTLFRDVFA 480
VL  Aphanocapsa GSE    ARKAQLGEQFEFDQETLSSDGILRTSPRGWFTYGHACFALLFFFGHIWHGARTLFRDVFS 480
VL  Pleurocapsa 7327   ARKAQLGESFDFDRETLNSDGVFRTSPRGWFTFAHACFALLFFFGHIWHGSRTLFRDVFA 480
VL  Fischerella 7521   ARKAQLGEVFEFDRETLNSDGVFRTSPRGWFTFGHACFALLFFFGHIWHGARTIYRDVFA 480
                       **:***** *.*::   .***::*:* **:*:: *. *.*::****:*** *::::***:

FRL Ancestral          GIDPDLDEEQVEFGVFQKVGDTTTRKKEAVAV---------------- 512
FRL Synechococcus 7335 GIDPSLSAEQVEWGYFKKVGDPTSQQTPA-------------------- 509
FRL Aphanocapsa GSE    GIDPELDEEQVEWGIFQKVGDKSSRKQRSLEPIPVPSASLPSQVSPQE 528
FRL Pleurocapsa 7327   GINPDLDEEQVEFGVWQKVGDVTSRKRQVA------------------ 510
FRL Fischerella 7521   GIDPELDE-QVEFGVFQKVGDTTTRKKQPVI----------------- 510
VL  Synechococcus 7335 GVDPDLSPQQVEWGFYQKVGDFSTKASK-------------------- 508
VL  Aphanocapsa GSE    GIDPELSEEQVEWGFYQKLGDTSTRRKETV------------------ 510
VL  Pleurocapsa 7327   GIDPDLG-EQVEFGLFAKVGDVSTRKEGV------------------- 508
VL  Fischerella 7521   GIDPDLE-EQVEFGVFAKVGDLSTRKKEAV------------------ 509
                       *::*.*    ***:* : *:** :::
```

PsbC

```
FRL Ancestral          METPFDRSVVTTETMSTTTIPDKTTKGTSITTAREGRDEASTGYAWWAGNARFINTELSG 60
FRL Synechococcus 7335 METPLETIP----D---LSLSPTAEVGSILAPASPGYDEATSGYAWWAGNARLITPELTG 53
FRL Aphanocapsa GSE    -----------------------------MTTSRDGRDEASTGYAWWAGNARFIN--LSG 29
FRL Pleurocapsa 7327   METPFNSSTVKIG----TTVQDAPTRDVTFDLAREGRDEASTGYAWWAGNARFIN--LSG 54
FRL Fischerella 7521   METPFDSKVSKPKD-------EVQKPAYIVNSPSEGRDEASTGYAWWAGNARFIN--QSG 51
VL  Synechococcus 7335 -----------------------MVTLSNNSFVGGGRDQPSTGYAWWSGNARLID--LSG 35
VL  Aphanocapsa GSE    -----------------------MVTLSSNSIVAGNRDQESSGFAWWAGNARLIN--LSG 35
VL  Pleurocapsa 7327   -----------------------MVTLSNTSYAGGGRDLSSTGFAWWAGNARLIN--LSG 35
VL  Fischerella 7521   -----------------------MVTLSRPGVLGAGRDQDSTGFAWWAGNARLIN--LSG 35
                                                    . *  ::*:***:****:*    :*

FRL Ancestral          RFLGAHVAHAGLIAFWAGAMLLFEVAHYVPEKPMYEQGLILMPHIATLGFGVGHGGEVVD 120
FRL Synechococcus 7335 RFLGAHVAHAGLVALWAGGMLLFEVSHFNLSKPMYEQGCILMPHIATLGIGVGQSGEITS 113
FRL Aphanocapsa GSE    RFLGAHIAHAGLMAFWAGAMLLFEVSHFVPEKPMYEQGLLLMPHVATLGFGVGPGGVVND 89
FRL Pleurocapsa 7327   KFLGAHVAHAGLIAFWAGAMLLFEVAHYVPEKPMYEQGCILMPHLATLGFGVGSGGQVVD 114
FRL Fischerella 7521   RFLGAHVAHAGLIAFWAGAMLLFEVAHYVPEKPMYDQGLILMPHIAALGFGVGPGGQVVD 111
VL  Synechococcus 7335 KLLGAHVAHAGLIVLWTGAMTLFEVSHYIPEKPMYEQGCILLPHLATLGWGVGPGGEVIN 95
VL  Aphanocapsa GSE    KLLGAHVAHAGLIVFWAGAMTLFEVAHFIPEKPMYEQGCILLPHIASLGWGVGPGGEVTD 95
VL  Pleurocapsa 7327   KLLGAHVAHAGLIVFWAGAMTLFEVAHFTPEKPMYEQGLILLPHLATLGWGVGPGGEVVD 95
VL  Fischerella 7521   KLLGAHVAHAGLIVFWAGAMTLFEVAHFVPEKPLYEQGLILLPHLATLGWGVGAGGEVVD 95
                       ::****:*****:.:*:*.* ****:*:  .**:*:** :*:**:*:** *** .* : .

FRL Ancestral          IFPFFAIAVIHLIGSAVLGFGGLYHSLRGPEKL---SGFFDFDWSDKDKITTILGYHLIA 177
FRL Synechococcus 7335 MFPFFAIGVAHLIGSAVLGIGGMYHAIKGPEKL---YGFFQFDWTDRAKVAQILGFHIAI 170
FRL Aphanocapsa GSE    IFPFFAIAVIHLIGSAVLGFGGVFHSIKGPAIL---PGYYDIDYADKDKITDILAYNLFW 146
FRL Pleurocapsa 7327   TFPYFAIAVIHLIGSAVLGIGGLYHSLRGPEKL---AGFFDFDWSDKDKMTSIIGYHLIA 171
FRL Fischerella 7521   IFPFFAIAVAHLIGSAVLGFGGIYHSLKGPQKL---PGFFNFDWSDKDKVTSILGYHLIA 168
VL  Synechococcus 7335 LFPYFVVGVLHLVSSAVLGLGGVYHALRGPETLEEYSSFFSQDWKDKNQMTNIIGYHLIL 155
VL  Aphanocapsa GSE    IFPFFVVGVVHLISSAVLGLGGIYHAVRGPEVLEEYSSFFGYDWKDKNQMTSILGFHLIV 155
VL  Pleurocapsa 7327   TFPYFVVGVLHLISSAVLGFGGIYHALRGPETLEEYSSFFGYDWKDKNQMTNIIGYHLIL 155
VL  Fischerella 7521   TYPYFVIGVLHLISSAVLGFGGIYHAIRGPEVLEEYSSFFGYDWKDKNKMTNIIGFHLII 155
                         :*:*.:.* **:.*****:**::*:::**  *     .::   *: *: ::: *:.:::

FRL Ancestral          LGIAALLLVCKAMFWGGLYDTWAPGGGDVRLITNPTLNPLVIFGYLFRSPFGGSGWIVSV 237
FRL Synechococcus 7335 LGIFALLFAAKAMYWGGLYDPWAPGGGDVRLVTNPTLDPRIIFGYLIKRPTGGEGWIVSV 230
FRL Aphanocapsa GSE    LGVGAWLLVGKAMLWGGLYDTWAPGGGDVRLISHPTLNPLTIFGYLVRSPYGQEGWIVGV 206
FRL Pleurocapsa 7327   LGIGAFLLVGKAMFWGGLYDTWLPGGGGVRLVTNPTLDPRVIFGYFFKSPLGGSGWIVSV 231
FRL Fischerella 7521   LGVAAFLLVGKAMLWGGLYDTWAPGGGGVRLVTNPTLDPRVIFGYLFKGFTGGAGNIASV 228
VL  Synechococcus 7335 LGLGAFLLVIKACFLGGVYDTWAPGGGDVRVITNPTLNPGVIFGYLASSPFGGEGWIVGV 215
VL  Aphanocapsa GSE    LGFGALLLVIKAMFVGGLYDTWAPGGGDVRLVTNPTLNPAVIFGYLVKSPFGGDGWAVSV 215
VL  Pleurocapsa 7327   LGFGALLLVFKAMFFGGVYDTWAPGGGDVRVITNPTLNPAVIFGYLIKAPFGGEGWIISV 215
VL  Fischerella 7521   LGLGAFLLVLKAMFFGGVYDTWAPGGGDVRVITNPTLNPAVIFGYLLKSPFGGDGWIIGV 215
                       **. * *:. **   **:** * ****.**::::***:*  ****:      *  *   .*

FRL Ancestral          NNLEDIVGGHIWMGSILIAGGIWHILTKPFKWTHKVFIWSGEAYLSQSLGNIAGQAFIAT 297
FRL Synechococcus 7335 NNLEDIIGGHIWIGCILIAGGIWHILVPPLRWTYNLFPWTGETYLSQSLGNVAGQAFIAA 290
FRL Aphanocapsa GSE    NNMEDLVGGHIWIGTILILGGIWHYFTTPSKWTHKVFTWSGEAYLAQSLGNVCGQALIAT 266
FRL Pleurocapsa 7327   DNLEDIVGGHIWMGGILIAGGIWHIFTKPWKWTDKVFIWSGEAYLSQSLGNIAGQAFIAA 291
FRL Fischerella 7521   DNLEDLVGGHIWIGSLLILGGIWHIVTKPFKWTHKAFIWSGEAYLSQSLGNVAGQAFIAT 288
VL  Synechococcus 7335 NNMEDIIGGHIWIGLICIFGGVFHILTKPFGWARRALIWNGEAYLSYSIGAVSLMAFICS 275
VL  Aphanocapsa GSE    DNLEDVVGGHIWIGLICIAGGIWHVLTKPFAWARRAFVWSGEAYLSYSLGALSLMAFIAT 275
VL  Pleurocapsa 7327   DNMEDIIGGHIWVGLICIAGGIWHILTKPFGWARRAFIWSGEAYLSYSLGALSLMGFIAS 275
VL  Fischerella 7521   DNMEDIIGGHIWVGLICIFGGIFHILTKPFGWARRAFIWSGEAYLSYSLGALSLMGFIAS 275
                       :*:**::*****:* : * **::* .. *  *: . : *.**:**: *:* :.  .:*.:

FRL Ancestral          CFIWFNNTAYPSEFYGPTAPEASQAQALTFLVRDQKLGANVASAQGPTGLGKYLMRSPTG 357
FRL Synechococcus 7335 AFIWFNNTAYPSVFYGPTVPESSQAQSFVFLMRDQGMGADVASAQGPTGLGKYLQRSPTG 350
FRL Aphanocapsa GSE    AFIWFNTTAYPSEFYGPTAPESSQAQALTFLVRDQSLGANVASAQGPTGLGKYLMRSPTG 326
FRL Pleurocapsa 7327   MFIWFNNTAYPSEFYGPTVPEASQAQALVFLARDQRLGADIGTAQSVTGLGKYLMRSPTG 351
FRL Fischerella 7521   MFIWFNNTAYPSEFYGPTVAESSNAQALVFLVRDQNLGANVASAQGPTGLGKYLMRSPTG 348
VL  Synechococcus 7335 CYVWFNNTAYPSEFYGPTNAEASQAQAMTFLVRDQRLGANIGSAQGPTGLGKYLMRSPTG 335
VL  Aphanocapsa GSE    CFVWFNNTVYPSEFYGPTGPEASQAQAMTFLVRDQRLGANVGSAQGPTGLGKYLMRSPTG 335
VL  Pleurocapsa 7327   VYVWFNNTAYPSEFYGPTGMEASQAQAFTFLVRDQRLGANIASAQGPTGLGKYLMRSPTG 335
VL  Fischerella 7521   CFVWFNNTAYPSEFYGPTNAEASQAQSFIFLVRDQKLGANVASAQGPTGLGKYLMRSPSG 335
                         ::***.*.*** *****   *:*:**:: ** ***  *:*::.:**. ******* ***:*

FRL Ancestral          EIIFGGETMRFWDFRGPWLEPLRGPNGLDLDKLQNDIQPWQIRRAAEYMTHAPLGSLNSV 417
FRL Synechococcus 7335 EIIFGGETMRFWDARAPWLEPLRGKNGLDLDKLQHDVQPWQLRRAAEYMTHSPIGSLNSV 410
```

```
FRL Aphanocapsa GSE    EIIFGGETMRFWDFRGPWLEPLRGPNGLDLDKLRYDVQPWQIRRAAEYMTHAPLGSLNSV 386
FRL Pleurocapsa 7327   EIIFGGETMRFWDFRGPWLEPLRGTNGLDLKKLQNDIQPWQIRRASEYMVHAPLGSINSV 411
FRL Fischerella 7521   EIIFGGETMRFWDVKAPWLEPLRGPNGLDIDKLQHDVQPWQIRRASEYMTHAPIGSLNSV 408
VL  Synechococcus 7335 EIIFGGETMRFWDFDGPWLAPLRGTNGLDLDKLKNDIQPWQVRRAAEYMTHAPNASINSV 395
VL  Aphanocapsa GSE    EIIFGGETMRFWDFRGPWLEPLRGPNGLDLNKIKNDIQPWQARRAAEYMTHAPLGSLNSV 395
VL  Pleurocapsa 7327   EIIFGGETMRFWDFRGPWLEPMRGPNGLDLDKLRNDVQPWQVRRAAEYMTHAPLGSLNSV 395
VL  Fischerella 7521   EIIFGGETMRFWDFRGPWLEPLRGPNGLDLDKIKNDVQPWQIRRASEYMTHAPNGSINSV 395
                       *************  .*** *:** ****:.*:: *:**** ***:***.*:* .*:***

FRL Ancestral          GGVATEINSFNFVSPRTWLAAAHFIFAFLFLVGHLWHAGRARAAAAGFERGIDREDEPVL 477
FRL Synechococcus 7335 AGLATESNAFNYVSPRTWLASAHFIFGFFFLVGHLWHAGRARAAAAGFETGLDREDEPVL 470
FRL Aphanocapsa GSE    GGVATEVNSFNFVSPRSWLAASHFVFAFLFLVGHLWHAGRSRAAAAGFARGIDPEDEPVL 446
FRL Pleurocapsa 7327   AGLATDVNSFNYVSPRTWMTASHLIFALFFLVGHWWHAGRARAAAAGFVRGINREDEPVR 471
FRL Fischerella 7521   GGLATELNSFNFVGPRAWLASAHFVFALLFLVGHLWHAGRARAAAAGFERGIDREDEPVL 468
VL  Synechococcus 7335 GGIITEVNSVNFVNPRQWLASFHFVMAFFFLVGHLWHAGRARAAEGGFERGLNREAEPVL 455
VL  Aphanocapsa GSE    GGVATEINSVNFVSPRSWLSTSHFVLAFFFLVGHLWHAGRARAAVAGFEKGIERETEPVL 455
VL  Pleurocapsa 7327   GGVITDVNSFNYVSPRAWLATSHFVLGFFFLVGHLWHAGRARAAAAGFEKGIDRETEPVL 455
VL  Fischerella 7521   GGVITEPNSFNYVNPRAWLATSHFVLAFFFLVGHLWHAGRARAAAGGFEKGIDRETEPVL 455
                       .*: *: *:.*:*.** *::: *:::.::***** *****:*** .**  *:: * ***

FRL Ancestral          SMPDLD------ 483
FRL Synechococcus 7335 SMAPIDPSLRSD 482
FRL Aphanocapsa GSE    SMADIDS----- 453
FRL Pleurocapsa 7327   SMGDID------ 477
FRL Fischerella 7521   SMPPLD------ 474
VL  Synechococcus 7335 SMPDLD------ 461
VL  Aphanocapsa GSE    SMPNLD------ 461
VL  Pleurocapsa 7327   AMPDLD------ 461
VL  Fischerella 7521   FMKDLD------ 461
                        *  :*
```

PsbD

```
FRL Ancestral          MTIAIGRASQQNRGWFSTLDDWLKRDRFVFIGWSGLLLFPCAYLAIGGWFTGTTFVTSWY 60
FRL Synechococcus 7335 MTITMGSL-GSARDWIKQLDDWLKRDRFVFIGWSGLLLFPCSFLAIGAWFTGTTFVTSWY 59
FRL Aphanocapsa GSE    MTIALKPARQ-WGDWVFTLDDWLKRDRFVFIGWSGLLLFPCSFLALGGWFTGTTFVSSWY 59
FRL Pleurocapsa 7327   MTIAITR-RQPTQGWFRTLDDWLKRERFVFIGWSGLLLFPCAYLAIGGWFTGTTFVTAWY 59
FRL Fischerella 7521   MTIAIRPSRTRGFEWFYVLDDWLKRDRFVFIGWSGLLLFPCAYLAIGGWFTGTTFVTSWY 60
VL  Synechococcus 7335 MTIAMGQA-PAARGRFDVLDDWLKRDRFVFVGWSGLLLFPCAFLAIGGWMTGTTFVTSWY 59
VL  Aphanocapsa GSE    MTIAVGRA-QPTRGWFDVLDDWLKRDRFVFVGWSGILLFPCAFLALGGWMTGTAFVSSWY 59
VL  Pleurocapsa 7327   MTIAMGRA-QAQRGWFDVLDDWLKRDRFVFIGWSGLLLFPCAYLALGGWLTGTTFVTSWY 59
VL  Fischerella 7521   MTIAIGR--STTRGWFDVLDDWLKRDRFVFVGWSGLLLFPCAYLALGGWLTGTTFVTSWY 58
                       ***::           .  *******:****:****:*****::**:*.*:***:**::**

FRL Ancestral          THGLASSYLEGCNFLTAAVSTPADSMGHSLLLLWGPEAQGDFTRWCQIGGLWTFVAFHGA 120
FRL Synechococcus 7335 THGLVSSYLEGCNFLTVAVSTPAESMGHSLLLLWGPEASGDFVRWCQIGGLWTFTALHGV 119
FRL Aphanocapsa GSE    SHGLATSYLEGCNFLTAAVSTPPDSMGHSLLLLWGPEAQGDFTRWCQLGGLWTFVALHGA 119
FRL Pleurocapsa 7327   THGLVSSYLEGCNVLTAAVSTPADSMGHSLLLLWGPEANWNFTRWCQIGGLWTFTALHGA 119
FRL Fischerella 7521   THGIVSSYLEGCNFLTAAVSTPADSMGHSLLFLWGPEAQGDFTRWCQIGGLWNFVALHGV 120
VL  Synechococcus 7335 THGLASSYLEGCNFLTVAVSTPADSMGHSLLLLWGPEAQGDFVRWCQIGGLWAFVALHGA 119
VL  Aphanocapsa GSE    THGLASSYLEGCNFLTVAVSTPANSMGHSLLLLWGPEAQGDFTRWCQLGGLWTFVALHGA 119
VL  Pleurocapsa 7327   THGLASSYLEGCNFLTVAVSTPADAFGHSLLFLWGPEAQGNFTRWCQIGGLWPFVALHGA 119
VL  Fischerella 7521   THGLASSYLEGCNFLTVAVSTPADAMGHSLLLLWGPEAQGDFTRWCQLGGLWTFVALHGA 118
                       :**:.:*******.**.***** :::*****:******. :*.****:**** *.*:**.

FRL Ancestral          LGLIGFMLRQIEIARLVGIRPYNAIAFSAPIAVFVSVFLIYPLGQSSWFFAPSFGVAAIF 180
FRL Synechococcus 7335 FGLIGFMLRQIEIARLVGIRPYNAIAFSAPIAVYCATFLIYPLGQSSWFFGPGFGVSAIF 179
FRL Aphanocapsa GSE    FSLIGFCLRQLEIARLIGVRPYNALAFSAPIAVYVSVFLMYPIGQSSWFFAPSFGVTAIF 179
FRL Pleurocapsa 7327   LGLMGFMLRQLEIARLVGVRPYNAIAFSAPIAVYVSVFLVYPLFQSSWFFAPSFGIAGIF 179
FRL Fischerella 7521   FGLIGFMLRQFEIARAVGVRPYNAIAFSAPISVFVSVFLIYPLGQSSWFFAPSFGVAAIF 180
VL  Synechococcus 7335 FGLIGFMLRQFEISRLVGIRPYNAIAFSGPIAVFVSVFLMYPLGQSSWFFAPSFGVAAIF 179
VL  Aphanocapsa GSE    FGLIGFMLRQFEIARLVGLRPYNAIAFSGPIAVFVSVFLMYPLGQSSWFFAPSFGVAGIF 179
VL  Pleurocapsa 7327   FGLIGFMLRQFEIARLVGIRPYNAIAFSAPIAVFVSVFLMYPLGQSSWFFAPSFGVAGIF 179
VL  Fischerella 7521   FGLIGFMLRQFEIARLVGIRPYNAIAFSAPIAVFVSVFLMYPLGQSSWFFAPSFGVAAIF 178
                       :.*:** ***:**:* :*:******:***.**:*: :.**:**: ******.*.**::.**

FRL Ancestral          RFLLFFQGFHNYTLNPFHMMGVAGVLGGALLCAIHGATVENTLFKDTKNFNTFRGFSPTQ 240
FRL Synechococcus 7335 RFLLFFQGFHNYTLNPFHMMGVTGVLGGALLCAIHGATVQNTLFRDNQSKNTFKGFSTDQ 239
FRL Aphanocapsa GSE    RFLLFFQGFHNYTLNPFHMMGVGGVLGGALLCAIHGATVENTLFEKTRGFNTFSGFSPTQ 239
FRL Pleurocapsa 7327   RFVLFFQGFHNYTNNPFHMMGVAGVLGGALLCAIHGATVQNTIFEDTKSFNTFGGFSPTQ 239
FRL Fischerella 7521   RFLLFFQAFHNYTLNPFHMMGVAGVLGGALLCAIHGATVENTLFRDTKSFNTFGGFSPTQ 240
```

```
VL  Synechococcus 7335 RFLLFLQGFHNWTLNPFHMMGVAGILGGALLCAIHGATVENTLFQDDENANTFRAFEPTQ 239
VL  Aphanocapsa GSE    RFILFFQGFHNWTLNPFHMMGVAGILGGALLCAIHGATVENTLFEDGEKSNTFSAFNPTQ 239
VL  Pleurocapsa 7327   RFILFVQGFHNFTLNPFHMMGVAGVLGGALLCAIHGATVENTLFQDSDQANTFRAFQPTQ 239
VL  Fischerella 7521   RFLLFLQGFHNWTLNPFHMMGVAGVLGGALLCAIHGATVENTLFEDGEGANTFRAFNPTQ 238
                       **:**.*.***:* ******** *:**************:**:*..    *** .*.  *

FRL Ancestral          AEETYSMVTANRFWSQIFGIAFSNKRWLHFFMLFVPVTGLWMSAIGMVGLAFNLRAYDFV 300
FRL Synechococcus 7335 GEETYSMVTANRFWSQIFGIAFSNKRWLHFFMLFVPVTGLWMSAIGMAGLAFNLRAYDFV 299
FRL Aphanocapsa GSE    AEETYSMVTANRFWSQIFGIAFSNKRWLHFFMLFVPVFGLWMSAIGMVGLAFNLRSYDFV 299
FRL Pleurocapsa 7327   EEETYSMVTANRFWSQIFGIAFSNKRWLHFFMLFVPVTGLWMSAIGMVGLAFNLRAYDFV 299
FRL Fischerella 7521   AEETYSFVTANRYWSQIFGIAFSNKRWLHFFMLFVPVTGLWMSSIGMVGLAFNLRAYDFV 300
VL  Synechococcus 7335 SEETYSMVTANRFWSQIFGIAFSNKRWLHFFMLFVPVTGLWMSSIGIVGLALNLRAYDFV 299
VL  Aphanocapsa GSE    AEETYSMVTANRFWSQIFGIAFSNKRWLHFFMLFVPVTGLWMASIGVVGLALNLRSYDFI 299
VL  Pleurocapsa 7327   AEETYSMVTANRFWSQIFGIAFSNKRWLHFFMLFVPVTGLWMASIGIIGIALNLRAYDFV 299
VL  Fischerella 7521   AEETYSMVTANRFWSQIFGIAFSNKRWLHFFMLFVPVTGLWMSAIGIVGLALNLRAYDFV 298
                       *****:*****:*********************** ****::**: *:*:***:***:

FRL Ancestral          SQELRAAEDPEFETFYTKNILLNEGLRAWMAPQDQPHEHFVFPEEVLPRGNAL- 353
FRL Synechococcus 7335 SQEIRAAEDPEFETFYTKNILLNEGLRAWLSEMDQPAKKFVFPDEVLPRGFSE- 352
FRL Aphanocapsa GSE    SQELRAAEDPEFETFYTKNILLNEGLRAWMAPEDQPHEHFVFPEEVLPRGNAL- 352
FRL Pleurocapsa 7327   SQELRAAEDPEFETFYTKNILLNEGLRAWMATMDQPHERFQFPDEVLPRGNAAK 353
FRL Fischerella 7521   SQEIRAAEDPEFETFYTKNILLNEGARAWMAAQDQPHEKFQFPEEVMPRGNAE- 353
VL  Synechococcus 7335 SQEIRAAEDPEFETFYTKNILLNEGIRAWLSPQDQPHEKFVFPEEVLPRGNAL- 352
VL  Aphanocapsa GSE    SQEIRAAEDPEFETFYTKNVLLNEGIRAWMAPQDQPHENLSFPEEVLPRGNAL- 352
VL  Pleurocapsa 7327   SQELRAAEDPEFETFYTKNILLNEGLRAWMAPQDQPHENFVFPEEVLPRGNAL- 352
VL  Fischerella 7521   SQELRAAEDPEFETFYTKNILLNEGIRAWMAPQDQPHEKFVFPE---------- 342
                       ***:**************:***** ***::  *** :.: **:
```

<u>PsbH</u>

```
FRL Ancestral          MQITNFQPKKVAPIQYLLKQLNSDSGKVVPGWGTTPLMAILMVLFFVFLLIILQIFNSSI 60
FRL Synechococcus 7335 -MRQKYVSNKAAPLQYPLRKLNSEAGKVVPGWGTAPLMGIMLIALLLFILTILQLYNGTV 59
FRL Aphanocapsa GSE    MEQPNFPTKKVAPLQYFFRQFNSDAGKVVRGWGTAPLMVVLMLLFLVFLLIILQIFNASI 60
FRL Pleurocapsa 7327   MQVRKFAPKKVAPLQYFFKRFNSQAGKVIPGWGTTPLMLALMLLFFFFLLMLLEIVNASI 60
FRL Fischerella 7521   MQSQNFQPKKVAPVQYLLKQLNTEAGKVTPGWGTTPLMAALMLLLFIFLLMILEIVNSSI 60
VL  Synechococcus 7335 -------MAQRTKLGNLLKPLNSEYGKVSPGWGTTPVMGVFMVLFLVFLLIILQLYNSSL 53
VL  Aphanocapsa GSE    -------MAKQTWLGDVLRPLNAEYGKVAPGWGTTPLMAVFMGLFLVFLLIILQIYNSSL 53
VL  Pleurocapsa 7327   -------MAQRTRLGDILRPLNSEYGKVVPGWGTTPLMGVFMLLFLVFLLIILQIYNSSL 53
VL  Fischerella 7521   -------MAQRTRLGDILRPLNSEYGKVAPGWGTTPLMAVFMGLFFVFLLIILQLYNKSI 53
                                : : :   :: :*:: ***  ****:*:*  ::  ::.*:* :*:: * ::

FRL Ancestral          LIEGVDIAVDWSSLSK-------------------------------------------- 76
FRL Synechococcus 7335 IVEGIDV----------------------------------------------------- 66
FRL Aphanocapsa GSE    LLEGIN--IDWSTLSDYAEVAASTPYGQGQFASTGTSIFIGLVVFALTCLALIFYGATTY 118
FRL Pleurocapsa 7327   QLEGID--VDWKSLSY-------------------------------------------- 74
FRL Fischerella 7521   LLEGINMS---------------------------------------------------- 68
VL  Synechococcus 7335 IINNVD--VDWRSLGN-------------------------------------------- 67
VL  Aphanocapsa GSE    ILDGVN--VTWKSFAG-------------------------------------------- 67
VL  Pleurocapsa 7327   LLEGFS--VDWRSLGQ-------------------------------------------- 67
VL  Fischerella 7521   IIQDVS--VDWRSLGR-------------------------------------------- 67
                        ::...

FRL Ancestral          ----- 76
FRL Synechococcus 7335 ----- 66
FRL Aphanocapsa GSE    PKDQR 123
FRL Pleurocapsa 7327   ----- 74
FRL Fischerella 7521   ----- 68
VL  Synechococcus 7335 ----- 67
VL  Aphanocapsa GSE    ----- 67
VL  Pleurocapsa 7327   ----- 67
VL  Fischerella 7521   ----- 67
```

Supplementary Figure S6. Alignments of ancestral FRL-PSII sequences and selected extant FRL- and VL-specific PSII sequences. The sequence with purple font (top) is the calculated ancestral FRL sequence. Sequences with dark red font are FRL-specific sequences from four extant FaRLiP-capable cyanobacteria (*Synechococcus* 7335, *Aphanocapsa* GSE, *Pleurocapsa* 7327, and *Fischerella* 7521). Sequences with black font are VL sequences from those organisms. A residue is highlighted yellow in the ancestral sequence if it is identical to the FRL sequences from extant cyanobacteria and dissimilar to the VL sequences. A residue is highlighted green in the *Synechococcus* 7335 sequences if it is identical to the other FRL sequences and dissimilar to the VL sequences. Thus, a residue position

that is highlighted yellow in the ancestral sequence and green in the *Synechococcus* 7335 sequence is likely conserved from the FRL-PSII ancestor that is found in extant FRL-PSII, implying important functional significance. Residues that are highlighted green in the *Synechococcus* 7335 sequence but the same site is not highlighted in the ancestral sequence are likely to be later adaptations, possibly enhancing FRL absorption. In the FRL-PSII sequences from *Synechococcus* 7335, regions that were not modeled in the cryo-EM structure (PDB 7SA3 [2]) are shaded grey. In the PsbH sequences, those residues that were not modeled due to low homology are also shaded grey. In all sequence alignments, the Clustal Omega [3] sequence conservation identifiers are shown below the alignment.

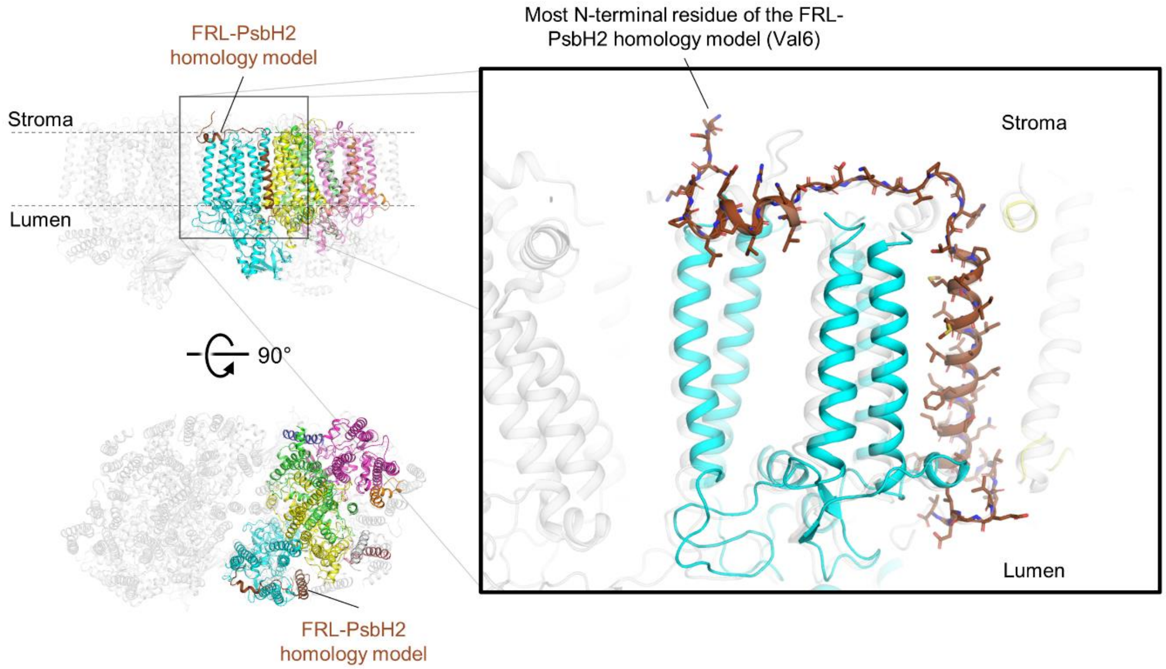

Supplementary Figure S7. Location of the FRL-PsbH2 homology model. On the left side, the *Synechococcus* 7335 apo-FRL-PSII structure is shown (colored) superimposed onto the structure of the PSII holocomplex from *Synechocystis* 6803 [5]. The FRL-PsbH homology model (brown) was generated using Swiss Model [6] and superimposed onto PsbH of the *Synechocystis* 6803 structure to approximate its location in the FRL-PSII complex. The magnification on the right shows the residues of FRL-PsbH additionally as sticks. The N-terminal region is shown extending across the stromal surface.

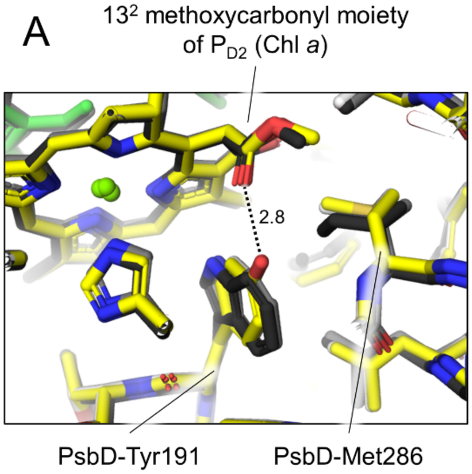

B

PsbD (D2) sequences

```
                                  |                 |
FRL Ancestral           FHNYTLN 195 ... AIGMVGL 290
FRL Synechococcus 7335  FHNYTLN 194 ... AIGMAGL 289
FRL Aphanocapsa GSE     FHNYTLN 194 ... AIGMVGL 289
FRL Pleurocapsa 7327    FHNYTNN 194 ... AIGMVGL 289
FRL Fischerella 7521    FHNYTLN 195 ... SIGMVGL 290
VL  Synechococcus 7335  FHNWTLN 194 ... SIGIVGL 289
VL  Aphanocapsa GSE     FHNWTLN 194 ... SIGVVGL 289
VL  Pleurocapsa 7327    FHNFTLN 194 ... SIGIIGI 289
VL  Fischerella 7521    FHNWTLN 193 ... AIGIVGL 288
                        ***:* *         **: *:*
```

Supplementary Figure S8. Conservation of FRL-PsbD interactions near P_{D2}. In (**A**), the *S.* 7335 apo-FRL-PSII structure (yellow), the homology model of the FRL-ASR (white), and two non-FaRLiP holocomplex PSII structures (light and dark grey from *T. vulcanus* and *Synechocystis* 6803, respectively) are superimposed. It also shows the H-bonding interaction involving the 13^2 methoxycarbonyl moiety with a dashed line with its distance in units of Å. In (**B**), two partial sequence alignments are shown that includes the FRL-ancestral sequence, FRL-specific sequences, and VL sequences from three FaRLiP-capable cyanobacteria. Conserved FRL-specific residues in extant cyanobacteria are highlighted in green in the sequence from *Synechococcus* 7335. If the same position is conserved in the FRL ancestral sequence, it is highlighted in yellow. Vertical lines above residue positions in (**B**) correspond to amino acids from the *Synechococcus* 7335 apo-FRL-PSII structure labeled in (**A**). The Clustal Omega [3] sequence conservation identifiers are shown below the alignment.

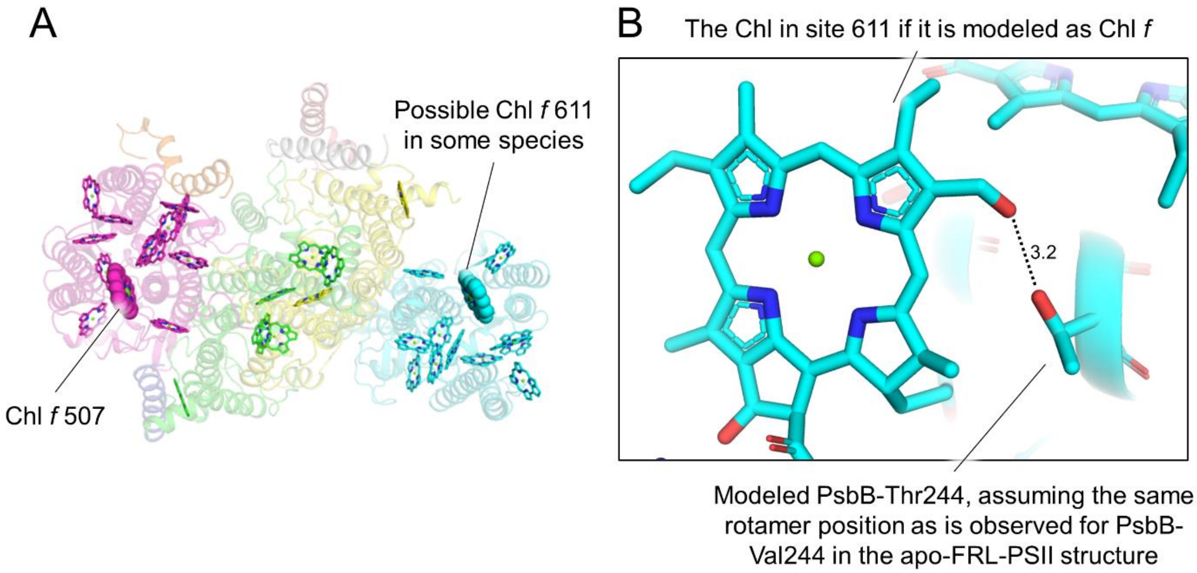

Supplementary Figure S9. Symmetry-related locations of Chl sites 507 and 611. In (**A**), a stromal view of the apo-FRL-PSII structure from *Synechococcus* 7335 is shown with transparent cartoons. Only Chl and pheophytin tetrapyrrole rings are shown, either as sticks, or spheres (507 and 611). In (**B**), the PsbB site 611 containing Chl *a* in the apo-FRL-PSII structure was instead fit with a Chl *f* molecule, and the Val in position 121 was instead modeled as a Thr with the same rotamer position. If the formyl moiety is directed toward the Thr sidechain, it is within H-bonding distance of the hydroxyl moiety of the Thr. The distance is shown in units of Å. Modeling was performed using Coot [4].

Supplementary Data

Supplementary Data S1. Sequence alignments with ancestral sequence reconstructions, and phylogenetic trees used in this study. (external)

Supplementary References

1. Hessa, T.; Meindl-Beinker, N.M.; Bernsel, A.; Kim, H.; Sato, Y.; Lerch-Bader, M.; Nilsson, I.; White, S.H.; von Heijne, G. Molecular Code for Transmembrane-Helix Recognition by the Sec61 Translocon. *Nature* **2007**, *450*, 1026–1030, doi:10.1038/nature06387.
2. Gisriel, C.J.; Shen, G.; Ho, M.-Y.; Kurashov, V.; Flesher, D.A.; Wang, J.; Armstrong, W.H.; Golbeck, J.H.; Gunner, M.R.; Vinyard, D.J.; et al. Structure of a Monomeric Photosystem II Core Complex from a Cyanobacterium Acclimated to Far-Red Light Reveals the Functions of Chlorophylls *d* and *f*. *J. Biol. Chem.* **2022**, *298*, 101424, doi:10.1016/j.jbc.2021.101424.
3. Sievers, F.; Wilm, A.; Dineen, D.; Gibson, T.J.; Karplus, K.; Li, W.; Lopez, R.; McWilliam, H.; Remmert, M.; Söding, J.; et al. Fast, Scalable Generation of High Quality Protein Multiple Sequence Alignments Using Clustal Omega. *Mol. Syst. Biol.* **2011**, *7*, 1–6.
4. Emsley, P.; Lohkamp, B.; Scott, W.G.; Cowtan, K. Features and Development of Coot. *Acta Crystallogr. Sect. D Biol. Crystallogr.* **2010**, *66*, 486–501, doi:10.1107/S0907444910007493.
5. Gisriel, C.J.; Wang, J.; Liu, J.; Flesher, D.A.; Reiss, K.M.; Huang, H.-L.; Yang, K.R.; Armstrong, W.H.; Gunner, M.R.; Batista, V.S.; et al. High-Resolution Cryo-EM Structure of Photosystem II from the Mesophilic Cyanobacterium, *Synechocystis* sp. PCC 6803. *Proc. Natl. Acad. Sci. USA* **2022**, *119*, e2116765118, doi:10.1073/pnas.2116765118.
6. Guex, N.; Peitsch, M.C.; Schwede, T. Automated Comparative Protein Structure Modeling with SWISS-MODEL and Swiss-PdbViewer: A Historical Perspective. *Electrophoresis* **2009**, *30*, S162–S173, doi:10.1002/elps.200900140.
